# Supplementary material for: Potential impacts of climate change on the productivity and soil carbon stocks of managed grasslands
Source: PLoS One. 2023 Apr 10;18(4):e0283370. doi: 10.1371/journal.pone.0283370 (PMC10085015; doi:10.1371/journal.pone.0283370)
Supplement: S1 File — (PDF) [file pone.0283370.s001.pdf]

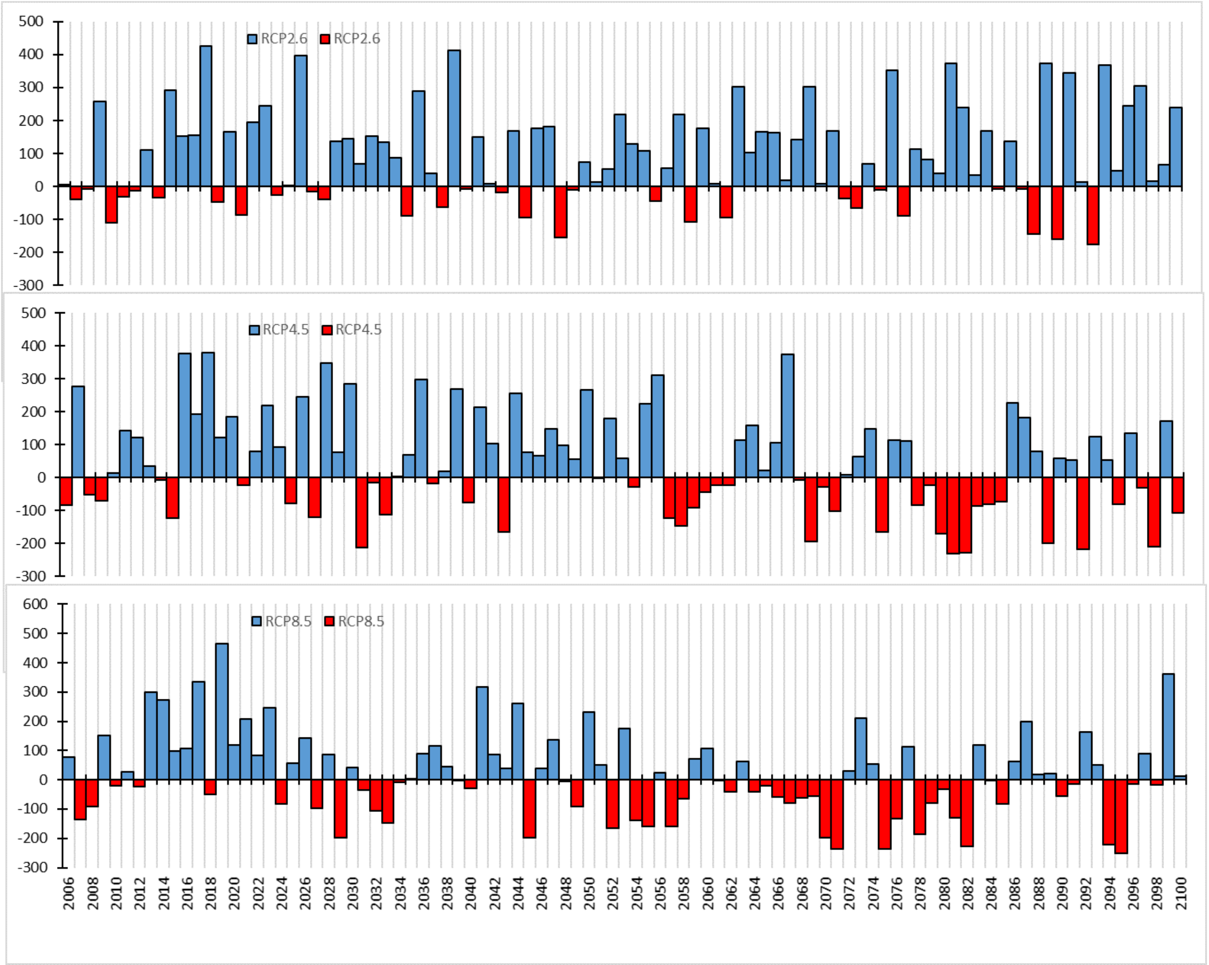


**Fig 1. Precipitation anomalies (mm) across the 21st century for three RCP scenarios downscaled for the Lusignan study site area.** Reference period span between 1975 and 2005.

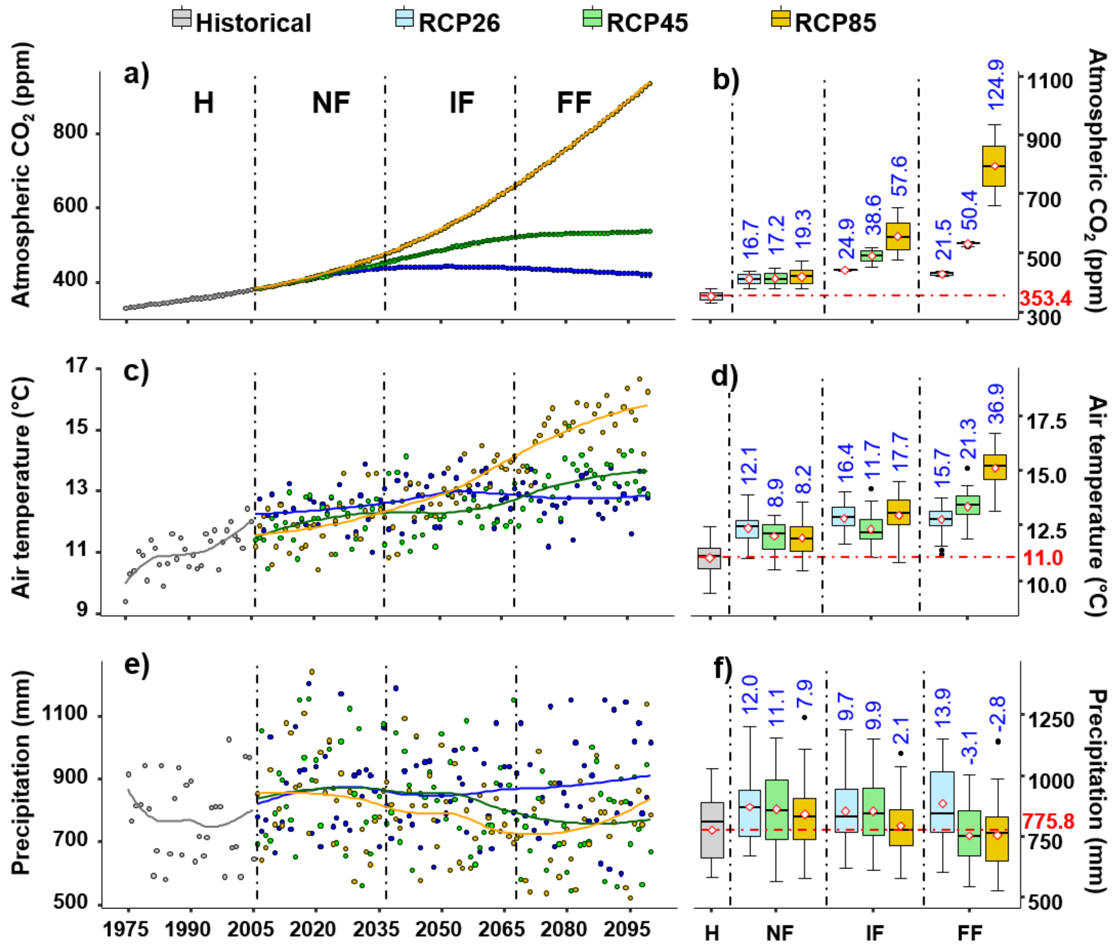


**Fig 2. Time series (a, c and e) and boxplots (b, d and f) of projected annual climate variables for 3 RCP scenarios (RCP2.6, RCP 4.5 and RCP 8.5) downscaled for Lusignan, France by the Aladin-climate model from CNRM. Atmospheric $CO_2$ concentration (a, b), air temperature (c, d) and annual precipitation (e, f).** Curves on time series graphs were obtained from non-parametric Loess smoothing. On boxplots, horizontal red dashed line and red numbers are the mean of the variable for the historical (H) period and blue numbers give the percent change of the variable mean in the different time horizons (near future: NF; intermediate future: IF; far future: FF) and for the different RCPs relative to the H period. The horizontal line within the boxes gives the median of observed values, diamonds give the averages, upper and lower sides of the boxes give the interquartile range between the 25$^{th}$ and 75$^{th}$ percentiles and the whiskers give the largest and lowest values within 1.5 times the interquartile range.

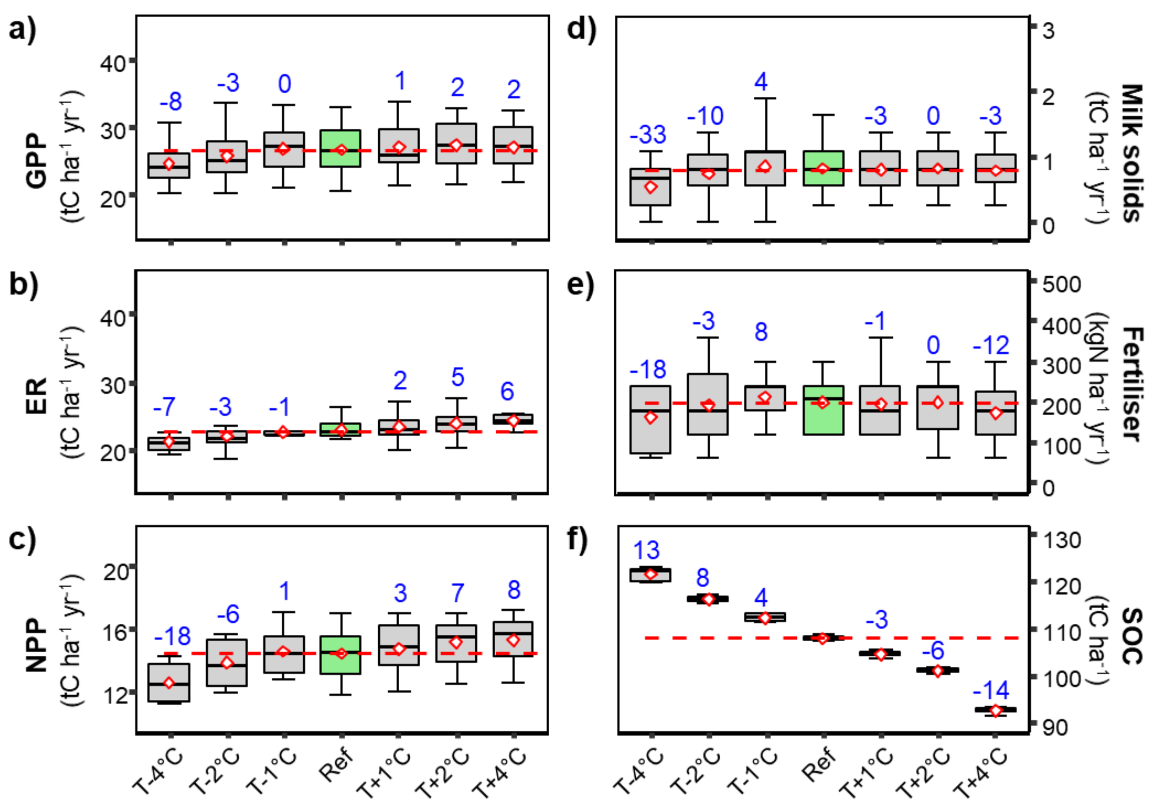


**Fig 3. Box and whiskers charts for modelled variables responses to changes in air temperature. Shown are modelled GPP a), ER b), NPP c), Milk-solids production d), N fertilizer e) and SOC stock f) responses to changes in air temperature for the mowed grassland.** Horizontal red dashed lines are the means of the variables for the reference period and blue numbers give the percentage changes of the variable means under the different changed meteorological conditions. The horizontal line within the boxes give the median of observed values, diamonds give the averages, upper and lower sides of the boxes give the interquartile range between the 25$^{th}$ and 75$^{th}$ percentiles and the whiskers give the largest and lowest values.

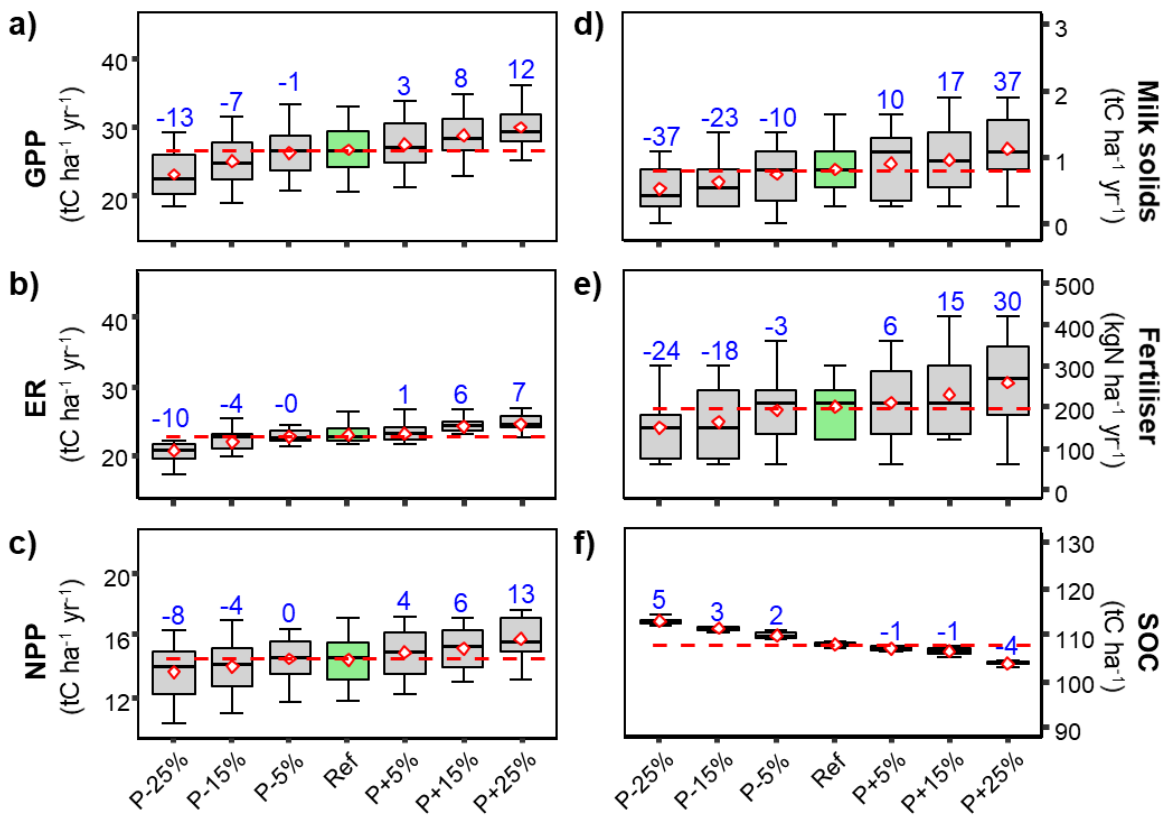


**Fig 4. Box and whiskers charts for modelled variables responses to changes in precipitation amounts. Shown are modelled GPP a), ER b), NPP c), Milk-solids production d), N fertilizer e) and SOC stock f) responses to changes in precipitation amounts for the <u>mowed</u> grassland.** Horizontal red dashed lines are the means of the variables for the reference period and blue numbers give the percentage changes of the variable means under the different changed meteorological conditions. The horizontal line within the boxes give the median of observed values, diamonds give the averages, upper and lower sides of the boxes give the interquartile range between the 25th and 75th percentiles and the whiskers give the largest and lowest values.

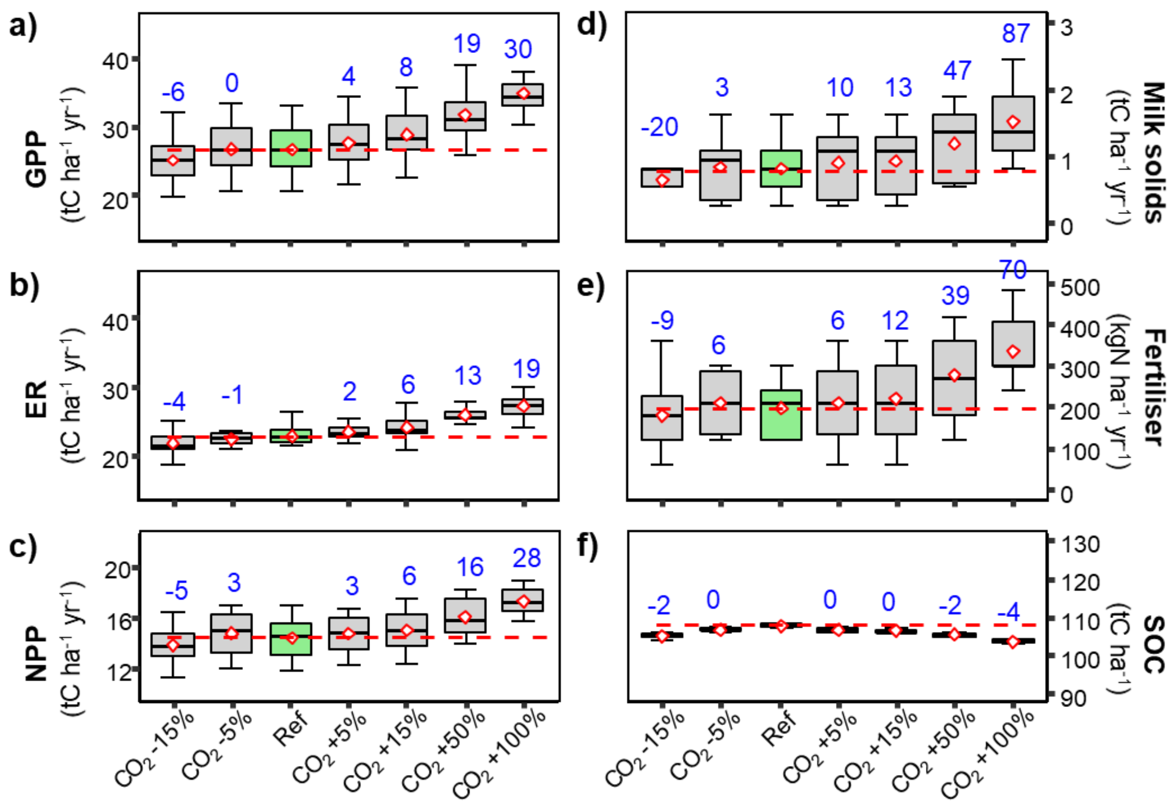


**Fig 5. Box and whiskers charts for modelled variables responses to changes in atmospheric $CO_2$ concentration. Shown are modelled GPP a), ER b), NPP c), Milk-solids production d), N fertilizer e) and SOC stock f) responses to changes in atmospheric $CO_2$ concentration for the mowed grassland.** Horizontal red dashed lines are the means of the variables for the reference period and blue numbers give the percentage changes of the variable means under the different changed meteorological conditions. The horizontal line within the boxes give the median of observed values, diamonds give the averages, upper and lower sides of the boxes give the interquartile range between the 25th and 75th percentiles and the whiskers give the largest and lowest values.

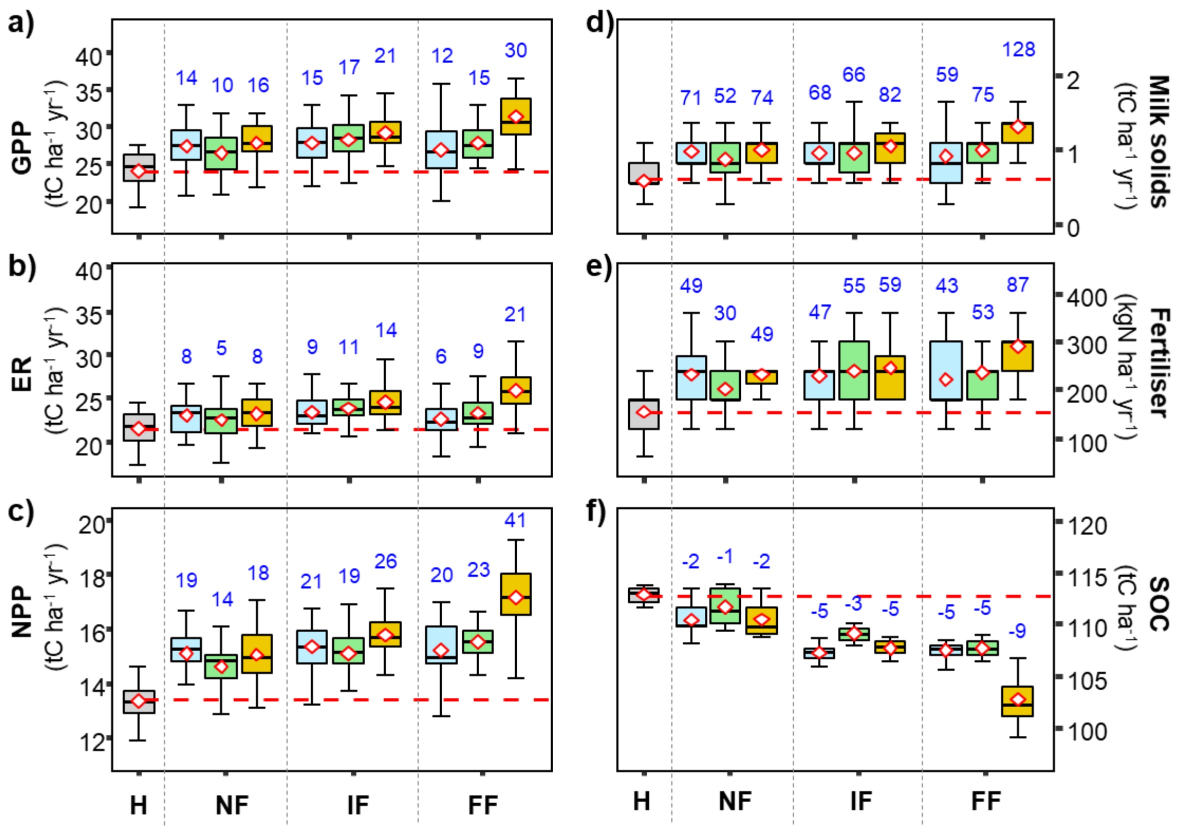


**Fig 6. Box and whiskers charts for modelled GPP a), ER b), NPP c), Milk-solids production d), N fertilizer e) and SOC stock f) for the mowed grassland site. Model runs were done under climate change projections under RCP 2.6 (blue), RCP 4.5 (green) and RCP 8.5 (orange) scenarios with their respective atmospheric $CO_2$ concentration dynamics.** Horizontal red dashed line and red numbers are the mean of the variable for the Historical (H) period and blue numbers give the percent change of the annual variables means for the different scenarios and time horizons.

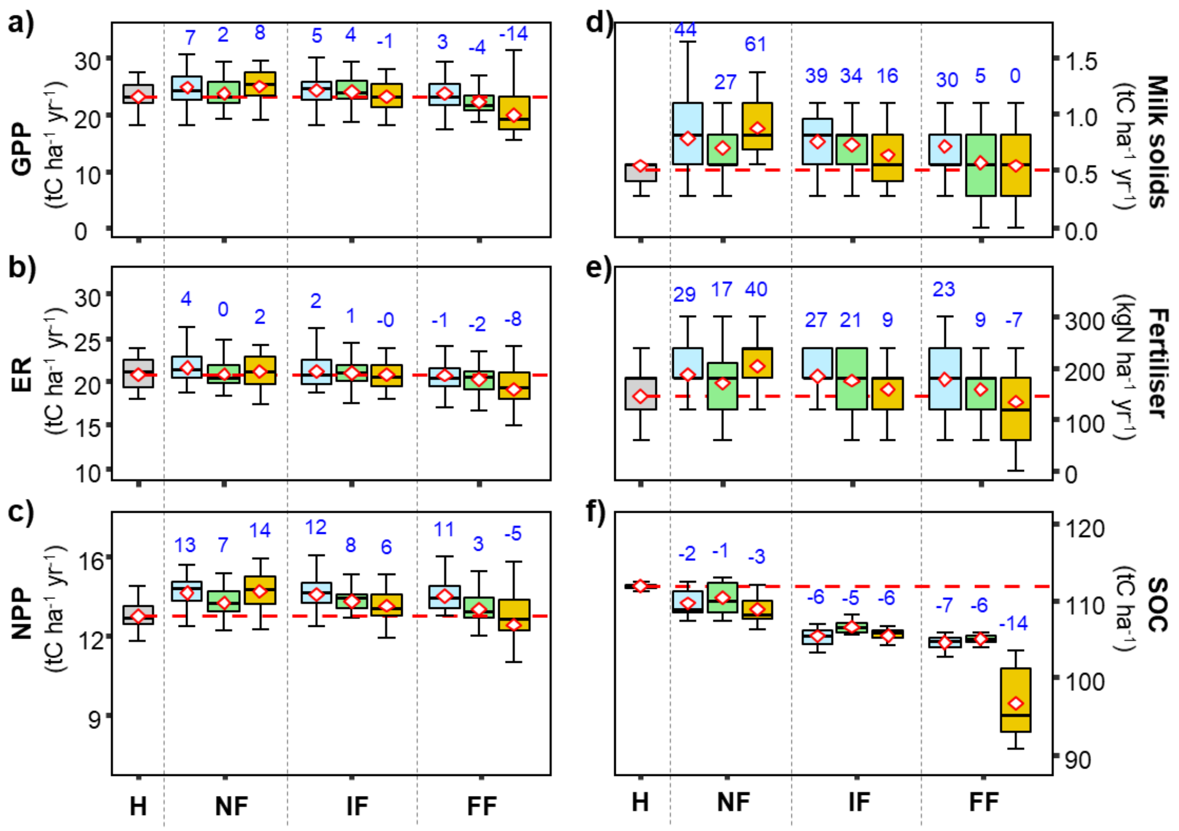


**Fig 7.: Box and whiskers charts for modelled GPP a, b), ER c, d), NPP e, f), Milk-solids production g, h), N fertilizer i, j) and SOC stock k, l) for the mowed grassland site. Model runs were done under climate change projections for RCP 2.6 (blue), RCP 4.5 (green) and RCP 8.5 (orange) with constant atmospheric $CO_2$ concentration equal to 380 ppm.** Horizontal red dashed line and red numbers are the means of the variables for the Historical (H) period and blue numbers give the percent change of the variable mean for the different scenarios and time horizons.

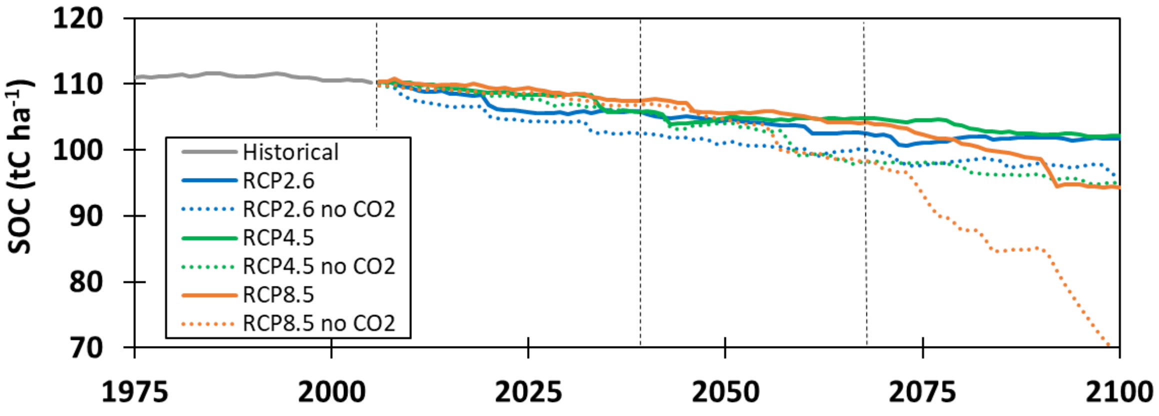


**Fig 8. Time series of modelled soil organic carbon (SOC) stocks modelled under RCP 2.6, RCP 4.5 and RCP 8.5 with and without $CO_2$ fertilization for the mowed paddock**

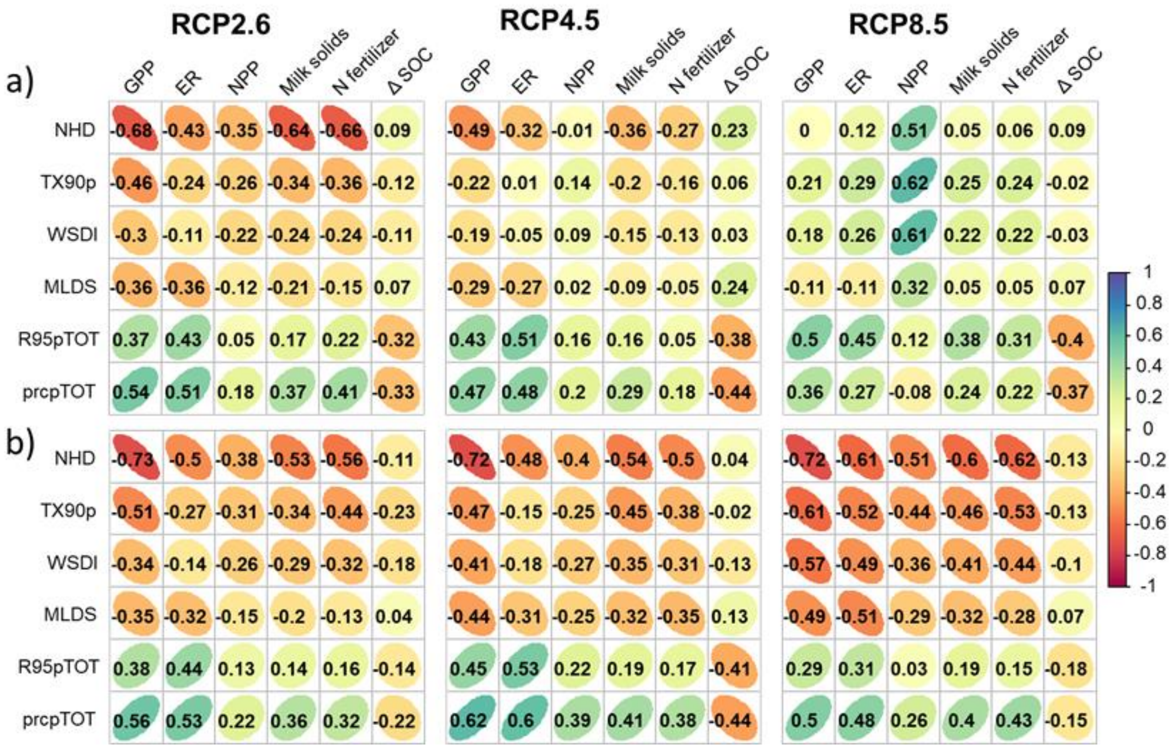


**Fig 9. Correlation matrixes between modelled GPP, ER, NPP, Milk solids, N fertilizer and change in SOC (ΔSOC) under RCP2.6, RCP4.5 and RCP8.5 climate conditions for mowing management with (a) and without (b) the $CO_2$ fertilization effect.** Numbers give the correlation coefficients and colors indicate correlation levels, going from red for negative to blue for positive correlations.

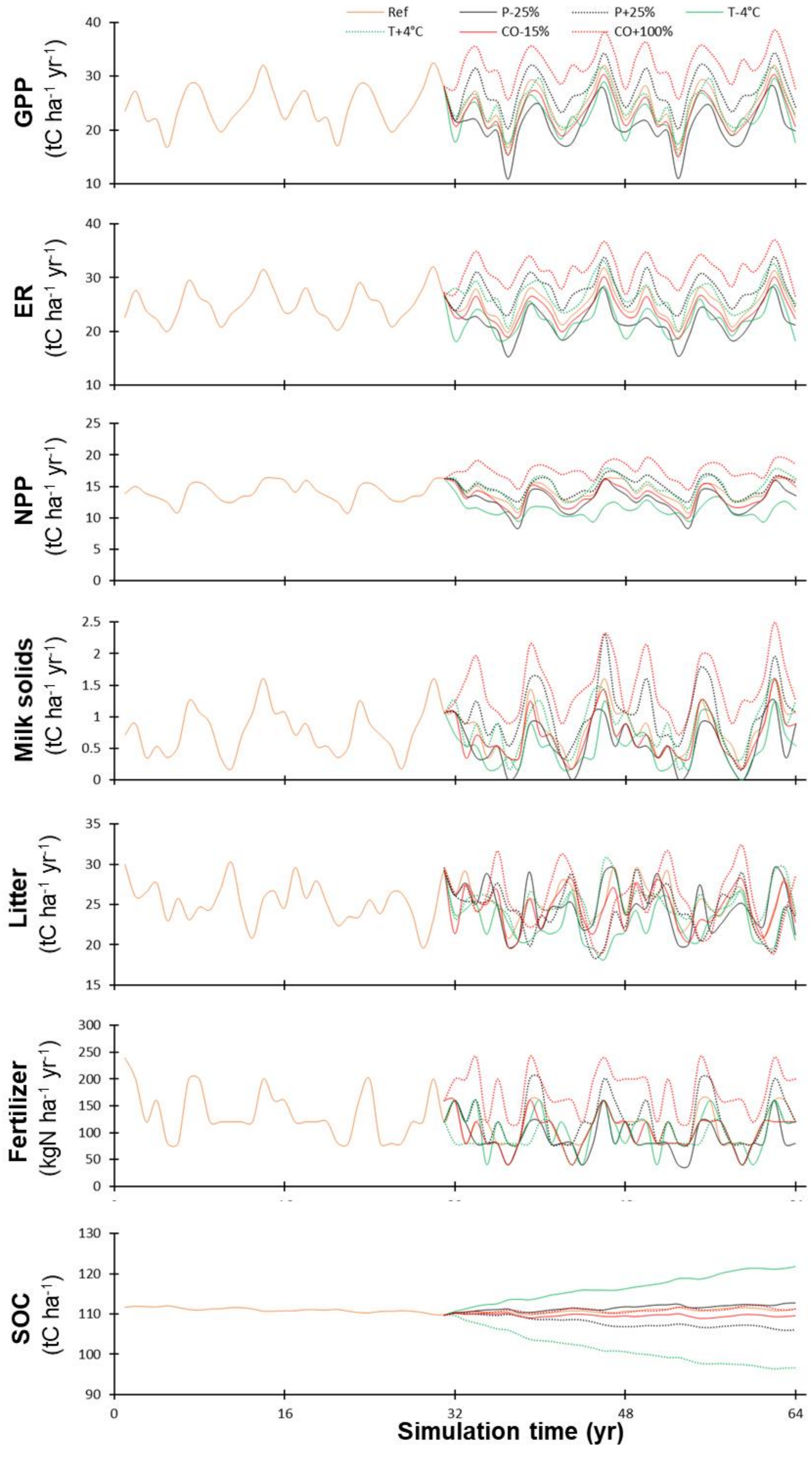


**Fig 10. Time series of modelled annual variables under the most extreme changes in meteorological conditions.**
