# Supplementary figures and images for: Potential impacts of climate change on the productivity and soil carbon stocks of managed grasslands
Source: PLoS One. 2023 Apr 10;18(4):e0283370. doi: 10.1371/journal.pone.0283370 (PMC10085015; doi:10.1371/journal.pone.0283370)

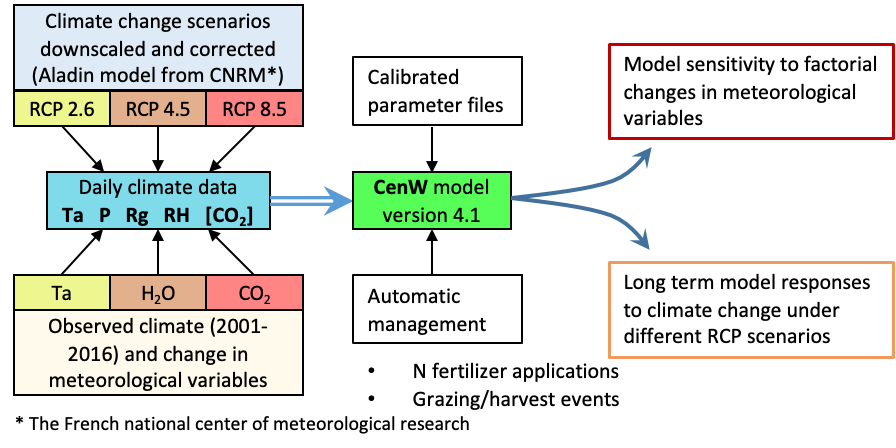

Supplement: S1 Graphical abstract — (TIF) [file pone.0283370.s002.tif]
