# Supplementary material for: Potential impacts of climate change on the productivity and soil carbon stocks of managed grasslands
Source: PLoS One. 2023 Apr 10;18(4):e0283370. doi: 10.1371/journal.pone.0283370 (PMC10085015; doi:10.1371/journal.pone.0283370)
Supplement: Attachment — Submitted filename: responses to reviewers010123.docx [file pone.0283370.s003.docx]

**Responses to Review Comments to the Author**

Response: We thank the reviewers and the editor for their time and helpful comments which certainly improved the quality of our work. In the following, we have provided responses to the reviewers' concerns about our manuscript.

**Reviewer #1**: The manuscript by Puche et al. investigate the impacts of the potential effects of future climatic conditions on productivity and soil organic carbon (SOC) stocks of mowed and rotationally grazed grasslands in France. They used the CenW ecosystem model to simulate carbon, water, and nitrogen cycles in response to changes in environmental drivers and management practices. The article addresses the importance of tailoring farming practices to achieve the dual goals of maintaining agricultural production while safeguarding soil C stocks. This manuscript is an interesting story. However, I do not consider the article ready for publication in its current state, and suggest a major revision, especially of the introduction, result and discussion section. These descriptions of these sections are too boring and should revise them to make clear. In addition, many sentences in this paper are too long and ambiguous. Please revise the long sentences to make it more clear and readily understandable.

Response: Thank you for your apprehension of our work. We have carefully addressed all concerns. Specifically, we have clarified, shortened and drastically condensed the text, as well as edited long and ambiguous sentences throughout the manuscript. As a result, the introduction, results and discussion sections are now straightforward and easy to follow.

1) The paragraphs of Introduction are too scattered and do not focus on major questions. For example, the first, second and third paragraph should be integrated in one paragraph, and focus on main problem that related to this topic. The Introduction also is not very informative in its current state, not explaining the important background to the reader. Considering my comments, the literature review should focus on effects of climate change on the productivity and soil carbon stocks.

Response: The introduction section has been revised in line with the reviewer's comments. The content has been reduced and shortened (e.g. >300 words have been removed compared to the original version) and a number of edits have been made, including the addition of new references as suggested.

2) The Results are not concise enough and are boring. I suggest that the results should concise and concentrate on major results. Please revise the results to make the results clearer.

Response: The whole "Results" section has been extensively revised in line with the reviewer's comment. The section is now much clearer and emphasises the main results.

3) The 4.2.1, 4.2.2, 4.2.3 in the Discussion section should change to 4.2, 4.3, 4.4. I suggest that there is no need for subheadings in Discussion.

The reviewer's suggestion has been considered and the sub-headings removed from the Discussion section.

4) The Summary and Conclusion is too long and not concise enough. Please revise this section to one paragraph and summarize the main results.

Response: The reviewer's suggestion has been addressed, by editing the Summary section. Regarding the conclusion, we felt that a formal section was not necessary as all the relevant text is already given in the Discussion, plus a few sentences that we added here and there as a final point at the end of the Discussion. The main contribution of this paper is not an overall answer (like "hypothesis accepted or rejected"), but the number of small individual observations and trends, like the responses to the three independent variables, and then the integration across RCPs and time slices. All these individual observations play an important role and all contribute to the overall finding.

5) The section of 2.2.3. Climate change scenarios is too long and should revise this section to make it clear and articulate.

Answer: We have revised this part of the manuscript. The language has been changed to be clearer and we have divided section 2.2.3 into three sections to make it clearer. The first section now focuses on the sensitivity of the model to weather, the second on the RCP climate change scenarios and the third subsection on extreme climate events.

6) Please check the tense and voice in the manuscript.

Response: Thank you, the tense and voice have been carefully checked and corrected throughout the manuscript.

7) Please check the punctuation, format and spacing throughout the manuscript.

Response: Thank you, we have corrected the punctuation, format and spacing throughout the manuscript.

***MINOR COMMENTS:***

Line 80-84: This sentence is ambiguous and redundant. Please revise it.

Response: This sentence was corrected accordingly – e.g. lines 74-81:

Line 115-119: This paragraph is necessary? I think this section is redundant and not strongly related to other parts. Please revise this section.

Response: Thank you for your comment, we removed this paragraph in the revised version of the manuscript.

Line 127-129: Please revise this sentence and this sentence is chaos.

Response: Thank you. This sentence has been corrected appropriately – e.g. lines 81-83.

Line 243: Revise this sentence

Response: The sentence was revised appropriately – e.g. lines 204-207.

Line 355-364: These two paragraphs are too boring and it is strange to introduce the invalid results. Delete these sections. Maybe these sections should merge into the result below.

Response: Answer: thank you for the suggestion: We deleted the second paragraph "In the following....., and their C stocks." but decided to maintain the first part of the section to explain how we decided to present the results (e.g. Lines 302-306)

Line 365-586: The results is too long and boring. Please revise these results.

Response: Thank you for your valuable comments which helped to improve the overall quality standard of the manuscript. We have completely redrafted and reorganised the results section; please see the revised version of the manuscript.

Line 598: “112 tC ha-1 yr-1” or “112 t C ha-1 yr-1”? The same format of units is same below.

Response: we corrected the format of units throughout the manuscript to “tC ha^-1^ yr^-1^”

Line 613-614: This sentence is strange and change the sentence structure.

Response: The sentence has been revised accordingly - Lines 558-559.

Line 664-665: This sentence is strange and revise it.

Response: the sentence was deleted

**Reviewer #2:** This is a very interesting study, which is very helpful for us to understand the impact of climate change.

Response: Thank you for this positive apprehension of our work.

The first half of the thesis is written in too much detail, which is more like a dissertation. I suggest that authors simplify some common-sense content，such as Line 120 "Grassland ecosystems cover about one quarter of the Earth’s total land area".

Response: thank you, we corrected and re-wrote the first half of the manuscript accordingly.

Materials and methods should also be modified more succinctly, and some details of model settings can be put in supplementary materials.

Response: thank you for your comment, we modified the materials and methods section and simplified the details of the model settings.

**Reviewer #3:** This paper touched a very nice topic on effects of grassland on climate change which is important and 100% fits the scope of Journal.

But it lacks novelty. They study is basically a model output extraction and visualization. The author just picked an already existing grassland models, simulate some variables under climate change scenarios and Climate sensitivity. Most of the results are just about percentage of change in the output variables he extracted from the model. Considering the fact that the author considered one GCM which is not enough, these outcomes are associated with a lot of uncertainty. The paper has a good base for a nice study development for example by adding spatial dimension or by adding more insights on possible management strategies or deriving components of grassland biomass changes. However, in the current status, the study lacks novelty. Although the writing of the paper was not too bad, but it still requires to be correct by a native. Some of the sentences were not clear to me (below comment). Additionally, some minor improvement is required which was not a minor problem in my opinion if the author justify the novelty of the work.

Response: We appreciate the valuable comments you provided on our manuscript. We believe that our study is important. We decided to study a particular area for which the model was specifically parameterised and where detailed management practices were available. We consider that our study, rather than a global study of grasslands from which considerable uncertainties could be realised from interpolating management practices, is also important to better understand how managed grasslands in a temperate region are likely to respond to climate change. We agree that the choice of a single GCM output for the climate change projection may have introduced errors, but we were only interested in trends over long periods (30 years) and this minimised errors in the simulations.

***Details comment.***

Lines 144: do you mean that “This study has been based on previous experiments”, the word elsewhere sound a bit abstract

Response: the sentence has been edited for clarity- (e.g., lines 116-117).

Lines 148-149. The reference citation does not seem to be consistent with the journal format. Please move the website to the bibliography

Response: The sentence has been corrected and the website reference to the bibliography deleted (e.g. Line 119).

Line 150: please be more specific with “over the study period”: which study period are you talking about.

Response: “over the study period” was replaced by “From 2006 to 2010” to be more specific.

Line 156: I do not understand this. Four blocks of five 0.4 ha plot.

Response: the sentence was corrected – eg., Lines 128-131.

Line 166-167. Varied between which values? Please be specific.

Response: Response: details of farm management practices are found in Puche et al., 2019 and summarised in lines 137-144.

Lines 206, how many mowing events per growing seasons? And at which time?

Response: as we explained in the manuscript, the model used automatic routines, and therefore the timing and amounts of biomass being harvested depends of the meteorological conditions and amounts of biomass present on the simulated paddocks.

In addition to the information provided in the lines 139-144 and as explained in the manuscript, the model uses automatic routines, and thus the timing and amounts of biomass harvested depend on of the meteorological conditions and the amounts of biomass present on the simulated paddocks.

Line 208: what do you mean by “to model the weather conditions”, you do not model weather condition

Response: We agree with the reviewer's comments that we did not model the weather conditions but modified the observed weather conditions at the site in our simulations. We have deleted the entire sentence from the manuscript and reorganised the entire section.

Lines 215-217. I do not understand this. How did you split five years into two weeks? If this is not the original work of authors, I do not see the necessity to putting material methods of others here.

Response: Thank you for your comment, this is related to our previous work in which the model was parameterised and validated with daily eddy covariance data for the mowed and grazed grasslands we used in this study.

I do not understand the necessity of figure 1. If it is the original results of the authors, it should go to results section. If it is from previous study, then why reference is missing and why do we need it. I even think putting in supplementary is not necessary

Response: thank you, we simplified the text of the section and removed the figure 1 from the manuscript.

Lines 237-238. I do not understand this. In lines 208 and 209 you mention 2005-2010. In lines 228, you said 2005-2016 and now here 2001-2016.

Response: thank you, the text was corrected.

Lines 241-260, I do not understand this. This section belongs to climate change. Or at least another additional section of scenario analysis I also do not understand why we need to have these scenarios of temperature and precipitation increase/decrease?

Response: we added a new section in the materials and methods for the sensitivity of the model to changes in individual climate variables. It is important to test the responses of the model and of the variables being modelled for the system to these changes in individual meteorological climate conditions.

The whole lines 263-270 could be removed. These are basic known info. OR at least lines 263-265” “The RCP scenarios……..globalization”. Consider removing this sentence.

Response: thank you, lines 263-265 were deleted as suggested by the reviewer (see lines 226-232)

271-272. all variables needed for running CenW.

Response: thank you, it is corrected

Line 271: explain CNRM2014 experiment.

Response: Details of the CNRM2014 experiment are given in the text – e.g. lines 235-241.”

Lines 277: why did you use abbreviation of RCM when you used this word only once?

Response: we agree with the reviewer, and we removed unnecessary abbreviations from the manuscript text.

Lines 339-343. Some of this paragraph, belongs to result section.

Response: we agree with the reviewer, and we removed unnecessary abbreviations from the manuscript text.

Lines 344-349: some of this belongs to results. Accordingly Tabl2 2 should be in result section.

Response: we followed the reviewer suggestion and we moved the Table 2 and the text to the result section.

The whole lines 355-364 is not necessary. This kind of information is usually helpful in report not scientific paper.

Response: Thank you, we removed the sentences to improve the quality of the publication.

Lines 366-367- Please consider removing. Lines 366-372 belongs to methodology. You should explain why these variables important. Just presenting model outputs does not justify this.

Response: we agree with the reviewer that presenting model outputs does not justify their use however we decided to keep these lines just as a reminder to the reader of what we will present in the result and discussion sections.

Lines 374-383. I would suggest to put results from both grazed and mowed grasslands here. One column for each in fig. 2. The same for figure 3

Response: thank you for your comment, we prefer to be consistent throughout the manuscript and present the results for the grazed grassland in the main text and the results for the mowed grassland in supplementary. As we mentioned the results for the two management practices gave very similar results.

I do not understand why we need a short section 3.1.1. In section 3.1.2 you still continue talking about the same figure. Why not putting everything in one section.

Response: thank you, we removed the short section 3.1.1

Lines 385-387. This is expected. Right?

Response: we agree with the reviewer, these results are expected and confirmed that the model was parameterised and responded properly to changes in meteorological conditions. This is very important if we want to run long-term simulations under climate change.

Lines 388-390. I did not understand this part. Please consider rephrasing.

Response: we simplified the presentation of the results and this sentence was removed because as we previously said no clear trends were identified.

Lines 410-411.”More specifically, in the years for which milk solids productions was reduced to zero (principally under T- 4°C and T-2°C), we carefully analyzed modelled and weather variables time series.” What do you mean? Why were you careful with this scenario and not with the others? You may see you explored the reason for reduced production.

Response: we thank the reviewer for pointing this error in the words that we used to present our results.

All the results have been carefully analysed, but we wanted to specify that we were looking for the causes of the decrease in productivity. We have reworded the sentence (e.g. 363-369) to avoid any confusion

Lines 424-426. What is novel about this result? This is general feedback. Are you expected something different from the model?

Response: we thank the reviewer for this comment. The reviewer is wright and these results confirmed that the model could simulate appropriately changes in annual precipitation amounts, which is important to analyse the responses of modelled variables under long-term climate change.

Lines 478-481- consider removing. Not necessary.

Response: we thank the reviewer for this comment. The sentence was removed

Lines 486-489: this belongs to discussion.

Response: we agree with the reviewer and the sentence was moved to the discussion section

Lines 508-510. This belongs to methodology.

Response: we agree with the reviewer and the sentence was moved to the materials and methods section
